# Remarkable response to radiation in a non-enhancing diffuse pediatric-type high-grade glioma with germline *ATM* mutation: The role of PET imaging and integrated histological and molecular analysis

**DOI:** 10.1093/noajnl/vdag059

**Published:** 2026-02-28

**Authors:** Barry Cheaney, Matthew D Wood, Laszlo Szidonya, Jana Ivanidze, Ali Nabavizadeh, Gagandeep Choudhary, Kimberly Wang, Maximilian Libmann, Josh Walker, Rebecca Ronsley, Erin Crotty, Matthew Miller, Sushant Puri, Aclan Dogan, Ramon F Barajas, Anh Huan Vo

**Affiliations:** Department of Neurological Surgery, Oregon Health & Science University, Portland (B.C., S.P., A.D., A.H.V.); Department of Pathology and Laboratory Medicine, Oregon Health & Science University, Portland (M.D.W.); Department of Radiology, Oregon Health & Science University, Portland (L.S., G.C., R.F.B.); Department of Radiology, Cornell University, New York City (J.I.); Department of Radiology, University of Pennsylvania, Philadelphia (A.N.); Department of Radiology, Oregon Health & Science University, Portland (L.S., G.C., R.F.B.); Department of Neurology, Oregon Health & Science University, Portland (K.W.); Department of Radiation Oncology, Oregon Health & Science University, Portland (J.W.); Ben Towne Center for Childhood Cancer and Blood Disorders Research and the Division of Hematology, Oncology, Bone Marrow Transplant & Cellular Therapy, Department of Pediatrics, Seattle Children’s Hospital, University of Washington, Seattle (R.R., E.C.); Ben Towne Center for Childhood Cancer and Blood Disorders Research and the Division of Hematology, Oncology, Bone Marrow Transplant & Cellular Therapy, Department of Pediatrics, Seattle Children’s Hospital, University of Washington, Seattle (R.R., E.C.); Department of Pediatrics, Oregon Health & Science University, Portland (M.M.); Department of Neurological Surgery, Oregon Health & Science University, Portland (B.C., S.P., A.D., A.H.V.); Department of Radiology, Oregon Health & Science University, Portland (L.S., G.C., R.F.B.); Knight Cancer Institute, Oregon Health & Science University, Portland (S.P., A.H.V., R.F.B.); Advanced Imaging Research Center, Oregon Health & Science University, Portland (R.F.B.); Department of Neurological Surgery, Oregon Health & Science University, Portland; Knight Cancer Institute, Oregon Health & Science University, Portland

**Keywords:** molecular analysis, neuro oncology, pediatric high grade glioma, PET MRI

## Abstract

We report a 19-year-old female with a non-enhancing, diffuse pediatric-type high-grade glioma, H3-wildtype and IDH-wildtype, harboring a germline *ATM* alteration. She presented with headaches, diplopia, papilledema, and obstructive hydrocephalus, and had a Karnofsky Performance Status of 70. MRI revealed infiltrative signal abnormality, and amino acid [F18]Fluciclovine PET/MRI identified increased avidity in the right lateral thalamus which guided biopsy sampling. Histopathology showed a hypercellular infiltrative glioma with high mitotic activity. DNA sequencing analysis with matched germline whole-exome sequencing identified a pathogenic *ATM* mutation, with biallelic *ATM* deficiency in tumor. Genome-wide DNA methylation confirmed the diagnosis. The patient received craniospinal radiation with a boost and concurrent temozolomide. She achieved rapid and significant neurologic recovery of function with KPS increasing to 90, accompanied by radiographic improvement. This case highlights the utility of PET/MRI in biopsy planning and underscores the importance of integrated histological and molecular diagnosis for management of non-enhancing gliomas.

Pediatric-type diffuse high-grade gliomas (pHGG) are aggressive central nervous system tumors comprising 9% of pediatric brain tumors, with five-year survival rates below 20%.^1^ While most demonstrate contrast enhancement on MRI, reflecting high cellularity, microvascular proliferation, and necrosis, a rare subset presents as non-enhancing lesions, complicating diagnosis and management. Advanced imaging and molecular diagnostics are essential in these cases.^2^ [F18]Fluciclovine PET, an amino acid-based radiotracer, can detect metabolically active tumor regions even when MRI lacks enhancement, aiding in delineation, biopsy guidance, and differentiation from treatment-related changes.^3^ Molecular techniques, including next-generation sequencing and DNA methylation profiling, have revolutionized pHGG classification by identifying key genetic and epigenetic alterations, informing prognosis, treatment, and surveillance strategies. Comprehensive testing can also reveal germline mutations relevant to patient care and family counseling. We report a 19-year-old with non-enhancing pHGG harboring a germline *ATM* mutation who showed a notable response to radiation, highlighting the value of PET imaging and molecular profiling.

## Case Presentation and Imaging

**Figure 1.**
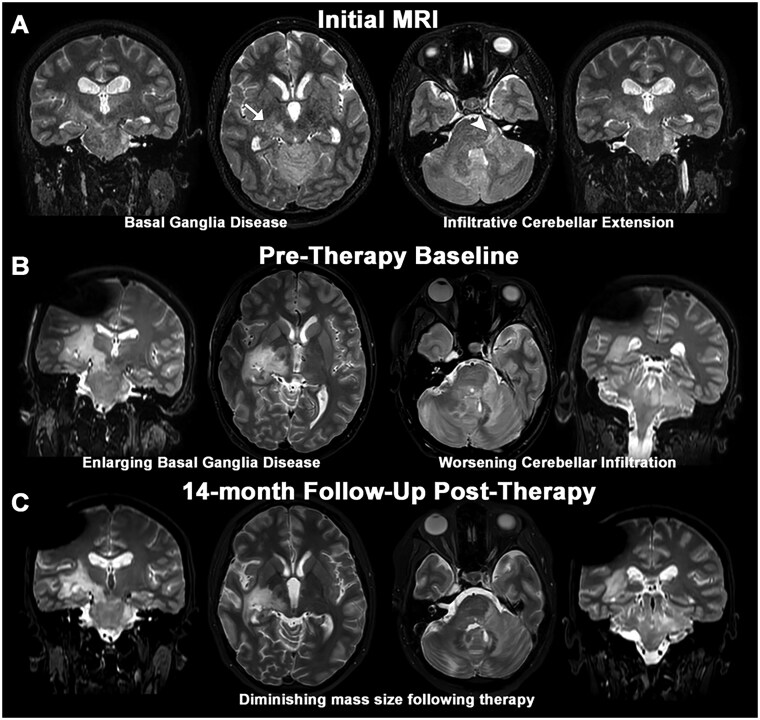
Longitudinal MRI assessment. (A) T2-weighted coronal (outside columns) and axial (inside columns) MRI from a 19-year-old woman who presents with an infiltrative homogenous non-enhancing T2 hyperintense mass centered within bilateral cerebellar hemispheres (arrow head), thalami (arrow), and brainstem, with discontinuous signal in the left amygdala. Volumetric analysis demonstrated a non-enhancing lesion volume of 8.05 mm^3^ (B) Baseline pre-therapy imaging 9-months after initial presentation shows nonspecific enlargement of the mass like T2-hyperintensity within a similar distribution (total volume 48.9 mm^3^; supratentorial component 23.0 mm^3^, infratentorial component 25.9 mm^3^). (C) Most recent follow-up imaging 14 months after initial presentation shows persistent, but decreased, appearance of the non-enhancing mass suggesting partial response to therapy Volumetric analysis demonstrated a non-enhancing lesion volume of 23.9 mm^3^. This was most prominent within the left cerebellar component (infratentorial lesion volume 6.0 mL).

**Figure 2.**
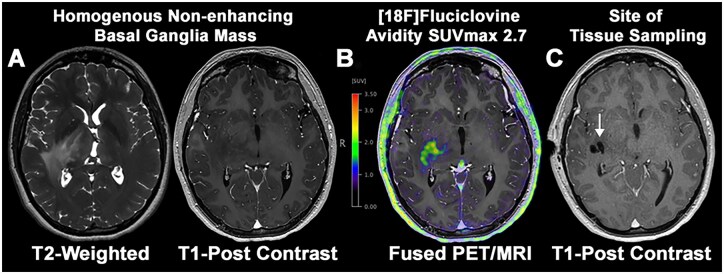
Amino acid [F18]Fluciclovine PET/MRI guides surgical tissue sampling. Multidisciplinary case review provided concern for primary glial neoplasm as a causative etiology of the non-enhancing lesion. This consensus opinion prompted the need for surgical tissue sampling as the next step in the patient’s care. However, the relatively homogenous MRI appearance of the non-enhancing lesion did not provide a definitive target. (A) Follow-up amino acid [F18]Fluciclovine PET/MRI surveillance imaging was performed 6 months after presentation to assess for elevated metabolism that could serve as a biopsy target. This demonstrated non-enhancing disease progression. (B) Elevated [F18]Fluciclovine avidity with an SUVmax of 2.7 was observed within the right basal ganglia that extended into the temporal stem. The [F18]Fluciclovine PET/MRI was integrated into the neuronavigational software and utilized to guide selection of a suitable tissue sampling target. (C) Post-surgical MRI demonstrates site of diagnostic tissue sampling corresponding to site of elevated [F18]Fluciclovine avidity (arrow).

This is a case of a 19-year-old female who presented with headaches for 6 months and 3 weeks of worsening diplopia found to have papilledema on ophthalmology evaluation. MRI revealed a non-enhancing infiltrative mass with heterogeneous T2 signal involving the bilateral cerebellar hemispheres, thalami, and brainstem, with discontinuous signal in the left amygdala (Figure 1A). There was no associated parenchymal diffusion restriction or increased relative cerebral blood volume by perfusion imaging. She underwent right frontal ventriculostomy placement due to her obstructive hydrocephalus and cerebrospinal fluid (CSF) was sent for infectious, inflammatory and cytology studies, all of which were negative. Her neurological status progressively declined over the subsequent six months. She was not established with the neuro-oncology team at this time. She developed new-onset left-sided numbness, decreased speed of finger tapping, orbiting movements around the left upper extremity, and a spastic gait requiring the use of assistive walking devices. Her visual symptoms worsened, with increasing diplopia and blurred vision. Follow-up surveillance MRI demonstrated interval disease progression, with extension of infiltrative signal abnormality into the right cerebral white matter (Figure 2A). Volumetric analysis demonstrated a non-enhancing lesion volume of 48.9 mm^3^ (supratentorial component 23.0 mL, infratentorial component 25.9 mL). Her Karnofsky Performance Status (KPS) had declined to 70 out of 100. She was subsequently referred to neuro-oncology for further evaluation and management. Advanced metabolic imaging with brain amino acid [F18]Fluciclovine PET/MRI was performed revealing an increased avidity up to SUVmax 2.75 in an expansile mass centered in the right lateral thalamus and posterior limb of the internal capsule, extending into the medial temporal stem. The temporal stem proved to be the area of highest SUVmax 2.25 avidity and was targeted for biopsy (Figure 2B and C).

## Pathology and Molecular Results

**Figure 3.**
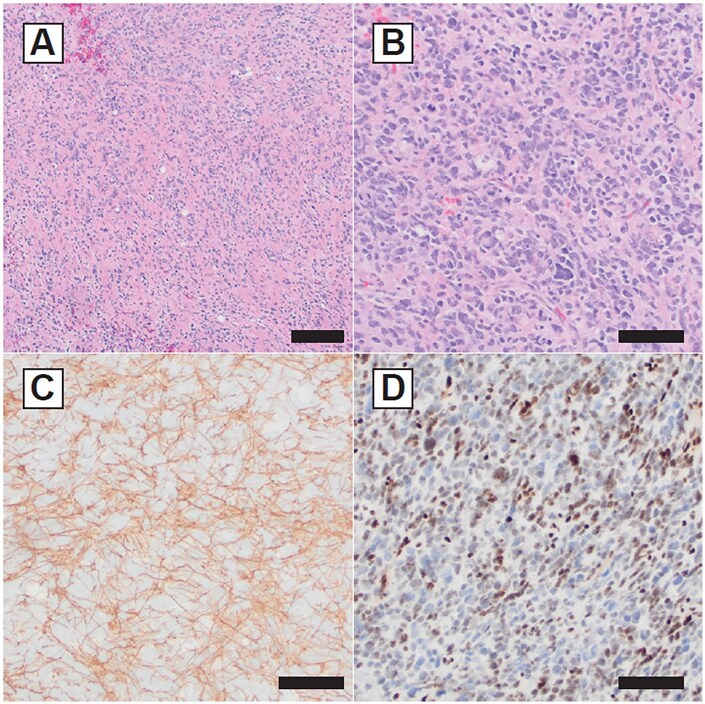
(A) Sections of the temporal lobe biopsy material showed fragments of a markedly hypercellular tumor infiltrating background brain parenchyma. (B) The tumor cells showed marked anisonucleosis, scant associated cytoplasm, and nuclear hyperchromasia without prominent nucleoli. (C) Immunohistochemical stains showed tumor-associated glial fibrillary processes. (D) An immunostain for trimethylated H3 lysine 27 (H3K27me3) showed a non-specific mosaic pattern of loss in tumor cells.

Sections of the temporal lobe biopsy material showed fragments of a markedly hypercellular tumor infiltrating background brain parenchyma (Figure 3A). The tumor cells showed marked anisonucleosis, scant associated cytoplasm, and nuclear hyperchromasia without prominent nucleoli (Figure 3B). Mitotic activity was high at 12 mitotic figures per mm^2^. Necrosis and microvascular proliferation were not identified. Immunohistochemical stains showed tumor-associated glial fibrillary processes with GFAP (Figure 3C). OLIG2 was negative in nearly all tumor cells and highlighted background/entrapped oligodendroglial cells. Immunostains for IDH1 R132H and H3 K27M (p.K28M) mutant proteins were negative. ATRX protein was retained, and p53 immunoreactivity was limited to scattered tumor cells only. INI1 and BRG1 protein immunoreactivities were intact. EZHIP protein was not over-expressed. An immunostain for trimethylated H3 lysine 27 (H3K27me3) showed a non-specific mosaic pattern of loss in tumor cells (Figure 3D). Overall, the histological and immunophenotypic features corresponded to a diffusely infiltrative high-grade central nervous system tumor, with differential including genetically and/or epigenetically defined types and subtypes of adult-type and pediatric-type diffuse high-grade glioma.

Molecular testing was performed at Nationwide Children’s Hospital through the Children’s Oncology Group’s Molecular Characterization Initiative for Childhood Cancers (MCI) in partnership with the CCDI (Childhood Cancer Data Initiative), which includes whole-exome sequencing of tumor tissue with matched germline comparator, RNA gene fusion testing, copy number analysis, and genome-wide tumor DNA methylation profiling. Somatic cancer-associated sequence variants were identified in *TP53* (p.Arg158Ser, NM_000546.6) and *SMARCA4* (p.Thr910Met, NM_003072.5). Numerous somatic cancer-associated copy number alterations and regions of loss of heterozygosity were identified, including relative copy number loss of 17p13.3-p13.1 encompassing the *TP53* locus and corresponding to an elevated variant allele frequency (VAF) of the *TP53* p. Arg158Ser variant of 89%. Testing of a germline (blood) sample identified a heterozygous intronic variant in *ATM*, c.5763-1056G>A (NM_000051.4, germline VAF 50%), that is predicted to result in a cryptic splice site leading to premature termination of translation^4^) In the tumor tissue, loss of heterozygosity of interstitial 11q was detected, resulting in biallelic *ATM* alteration in the patient’s tumor (tumor VAF 95% for *ATM* c.5763-1056G>A). The copy-number profile, included in Supplemental Table 1, demonstrates multiple relative whole-arm gains and losses. No gene fusions were detected. Classification of the patient’s tumor by genome-wide DNA methylation profiling using a classifier developed by the Institute for Genomic Medicine at Nationwide Children’s Hospital resulted high calibrated scores to superfamily pediatric-type diffuse high-grade glioma, family/class/subclass diffuse pediatric-type high-grade glioma, H3-wildtype and IDH-wildtype (all calibrated scores >0.99). MGMT promoter methylation status was unmethylated. Incorporating the histopathological, genetic, epigenetic, and germline findings resulted in an integrated diagnosis of diffuse pediatric-type high-grade glioma, IDH-wildtype and H3-wildtype, CNS WHO grade 4, arising in the setting of germline *ATM* alteration. Details of molecular testing results are provided as Supplemental Table 2.

## Treatment and Response

She was initiated on temozolomide at 75 mg/m^2^/day with 36 Gy craniospinal radiation therapy with a boost to posterior fossa and involved adjacent structures to 59.4 Gy in 1.8 Gy fractions started 6 weeks after her biopsy procedure. Baseline pre-radiotherapy MRI demonstrated enlargement of the non-enhancing mass (Figure 1B). Temozolomide was discontinued after 18 doses due to CTCAE version 5 grade 1 thrombocytopenia and grade 2 neutropenia. Remarkably, following completion of radiotherapy, the patient exhibited significant clinical improvement. She regained independent ambulation, with her most recent KPS improving to 90 out of 100. Her left-sided numbness had resolved completely. Although she continued to experience persistent diplopia, her overall functional capacity improved notably, and she reported engaging in swimming three times per week as part of her physical activity routine. Post-treatment MRI demonstrated an excellent radiographic response, with a marked decrease in the extent and intensity of previously noted infiltrative abnormalities (Figure 1C). Volumetric analysis demonstrated a non-enhancing lesion volume of 23.9 mm^3^ (supratentorial component 17.9 mL, infratentorial component 6.0 mL). The marked decrease in non-enhancing lesion size was favored to represent the sequela of tumor cytoreduction from efficacious therapy. While decreased vasogenic edema may have been a contributing factor, this was thought less of a contributing factor given the absence of bevacizumab therapy. However, this distinction was difficult to definitively assess given the absence of a clinical indication to perform follow-up [F18]Fluciclovine PET/MRI.

## Discussion

We present a compelling case in which image-guided biopsy and integrated histological-molecular analysis contributed to a dramatic positive clinical outcome in a patient with an *ATM*-deficient pHGG. This case demonstrates the utility of [F18]Fluciclovine PET/MRI in determining a safe and viable target for biopsy in the absence of a clear enhancing component. While no prior studies have specifically linked [F18]Fluciclovine PET/MRI findings in pediatric high-grade glioma in the setting of germline *ATM* mutations, our case highlights the diagnostic and procedural value of PET/MRI in evaluating a tumor in critical anatomic areas without contrast enhancement. In our patient, [F18]Fluciclovine uptake was confined to a subregion of the tumor despite multifocal T2-hyperintense abnormalities on MRI, reflecting the inherent metabolic heterogeneity of diffuse gliomas. Several mechanisms may account for this focal avidity. First, areas of higher cellular density and mitotic activity tend to overexpress amino acid transporters, leading to increased tracer accumulation. Second, fluciclovine transport is mediated primarily by LAT1 and ASCT2, whose expression is not uniform across gliomas and may generate metabolic “hotspots.” Third, tumor compartments may differ in fuel preference: regions enriched in glutamine or branched-chain amino acids may accumulate fluciclovine, while other portions of the tumor rely more heavily on glucose, acetate, or lipid metabolism. Fourth, local microenvironmental factors such as hypoxia, vascular supply, and nutrient gradients strongly influence pathway selection, with oxygenated regions engaging amino acid metabolism more than hypoxic zones. Finally, tumor evolution and subclonal genetics, including alterations in *TP53, SMARCA4*, or *ATM*, may further shape transporter expression and metabolic dependencies. Together, these factors highlight that focal fluciclovine uptake can identify the biologically most active compartment within an otherwise homogeneous-appearing lesion, underscoring the value of amino acid PET in guiding biopsy and treatment strategies.

*ATM* encodes a key kinase in the DNA damage response, orchestrating repair of double-strand DNA breaks through homologous recombination following cell-cycle checkpoint activation.^5^ Loss-of-function *ATM* mutations disrupt this repair network, rendering tumor cells particularly susceptible to DNA-damaging agents such as radiation, a phenomenon known as synthetic lethality.^6^^,^^7^ Preclinical studies have shown *ATM*-deficient cells exhibit marked radiosensitivity due to impaired repair of radiation-induced double-strand breaks and consequent genomic instability.^5^ Ataxia-telangiectasia (AT) is a rare autosomal recessive disorder caused by biallelic pathogenic variants in the *ATM* gene, characterized by progressive neurodegeneration, immunodeficiency, telangiectasias, and a high predisposition to malignancy and radiation sensitivity due to impaired DNA double-strand break repair.^8^ However, individuals who are heterozygous carriers of a single pathogenic *ATM* variant do not manifest the classical features of AT but nevertheless face elevated lifetime risks for various cancers, particularly breast, pancreatic, prostate, and gastric cancers.^9^ Moreover, recent data suggest heterozygous carriers may experience increased susceptibility to treatment-related toxicities, including heightened rates of myelosuppression during chemotherapy and radiation therapy, likely reflecting partial defects in DNA repair pathways.^10^ In our case, the patient carried a heterozygous germline *ATM* mutation, with tumor-specific loss of heterozygosity resulting in biallelic *ATM* inactivation within the tumor. Heterozygous germline mutations in the *ATM* gene occur in 0.5%-1% of the population and are associated with tumor predisposition.^9^ Notably, despite receiving aggressive craniospinal radiation therapy, she tolerated treatment well and demonstrated significant clinical recovery and radiographic improvement on follow-up imaging. These findings underscore the importance of recognizing *ATM* heterozygosity not only as a possible cancer predisposition state, but also as a factor influencing treatment tolerance and therapeutic decision-making in patients with high-grade gliomas, particularly when there is inactivation of the wildtype *ATM* allele in the tumor.

While germline *ATM* mutation has been identified as predisposing in groups of pediatric glioma cases and datasets, no detailed case report solely focusing on an individual with germline *ATM* presenting with pHGG has been described.^11^ Our patient is currently enrolled in an intraventricular CAR T-cell therapy clinical trial (NCT05768880), which represents an exciting and promising avenue for pediatric high-grade glioma. CAR T-cells can be engineered to specifically target tumor-associated antigens, potentially overcoming the limitations of conventional therapies in infiltrative and non-enhancing tumors.^12^ Future treatment options may include poly (ADP-ribose) polymerase inhibitors (PARPi), which impair DNA damage repair pathways and have demonstrated efficacy in tumors with *ATM* mutations, both as standalone therapy and in combination with temozolomide especially when there is biallelic loss of *ATM*.^13^ Notably, emerging data suggest these agents may be especially beneficial in MGMT-unmethylated tumors.^13^ Additionally, in pediatric high-grade gliomas, biallelic loss of *ATM* may induce a state of synthetic lethality, making these tumors potentially susceptible to ATR inhibitors, which target compensatory DNA repair pathways.^14^ While early phase pediatric studies have not clearly demonstrated an efficacy signal in using PARP inhibitors in solid malignancies, including central nervous system tumors, these trials were not enriched for tumors harboring biallelic *ATM* loss.^15^ Recent studies have demonstrated improved radiotherapy outcomes in *ATM*-mutant gliomas as well, supporting the notion that *ATM* deficiency enhances treatment sensitivity.^6^^,^^7^ In line with these observations, we hypothesize that our patient’s remarkable clinical and radiographic response to radiation therapy is attributable, at least in part, to the presence of a germline *ATM* mutation and subsequent biallelic loss within the tumor. Given the limitation of a single case report, these observations warrant future studies. Radiosensitivity can also be influenced in part by tumor biology (TP53, SMARCA4, or epigenetic signatures). This case emphasizes the need for complete molecular analysis on all pediatric and young adult patients with HGG including germline testing.

## Supplementary Material

vdag059_Supplementary_Data

## Data Availability

The data underlying this article are available in the article and in its online supplementary material.
