# Supplementary material for: Remarkable response to radiation in a non-enhancing diffuse pediatric-type high-grade glioma with germline ATM mutation: The role of PET imaging and integrated histological and molecular analysis
Source: Neurooncol Adv. 2026 Feb 28;8(1):vdag059. doi: 10.1093/noajnl/vdag059 (PMC13007283; doi:10.1093/noajnl/vdag059)
Supplement: vdag059_Supplementary_Data [file vdag059_supplementary_data.zip › Supp table 1.docx]

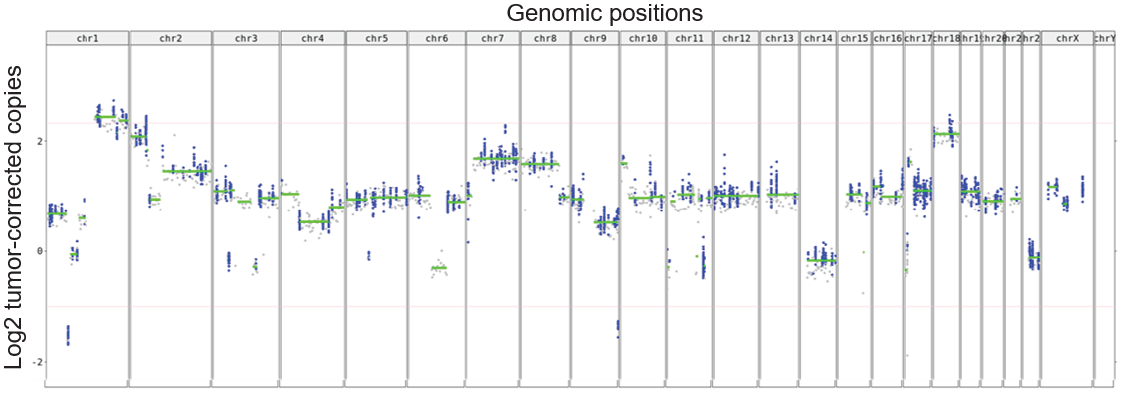


| **Cytogenetic Locus** | **Size (Mb)** | **Alteration** |
| --- | --- | --- |
| 1p36.33-p11.2 | 120.399 | Relative Whole Arm Loss |
| 1p11.2-q44 | 127.545 | Relative Whole Arm Gain |
| 2q11.1-q37.3 | 147.002 | Relative Whole Arm Gain |
| 7p21.1-q36.3 | 141.874 | Relative Gain |
| 11q22.3-q23.2 | 7.865 | Relative Loss with LOH |
| 14q11.2-q32.33 | 86.881 | Relative Whole Arm Loss with LOH |
| 17p13.3-p13.1 | 9.844 | Relative Loss with LOH |
| 22q11.1-q13.33 | 25.251 | Relative Whole Arm Loss with LOH |

**Supplemental Table 1.** Copy number profile from institutional next-generation DNA sequencing results (GeneTrails Comprehensive Solid Tumor Panel) and the corresponding somatic cancer-associated copy number variations and loss of heterozygosity in tumor reported by the reference laboratory testing (Molecular Characterization Initiative, Nationwide Children’s Hospital Institute for Genomic Medicine).
